# Supplementary material for: Causal Relationship between Aging and Anorexia Nervosa: A White-Matter-Microstructure-Mediated Mendelian Randomization Analysis
Source: Biomedicines. 2024 Aug 16;12(8):1874. doi: 10.3390/biomedicines12081874 (PMC11351342; doi:10.3390/biomedicines12081874)
Supplement: Supplementary file 1 [file biomedicines-12-01874-s001.zip › Supplementary Figure/Figure S4/Singe SNP plot of MR analysis for TL to ubm-b-1841.pdf]

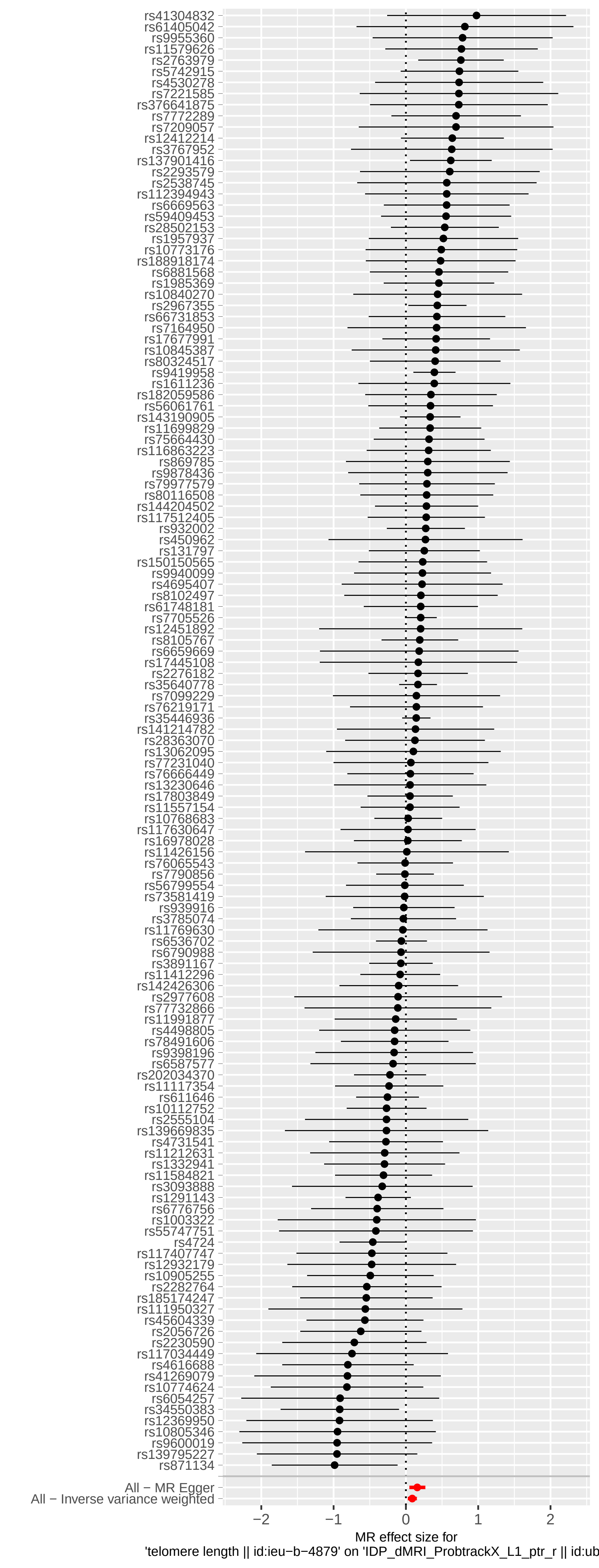
rs41304832
rs61405042
rs9955360
rs11579626
rs2763979
rs5742915
rs4530278
rs7221585
rs376641875
rs7772289
rs7209057
rs12412214
rs3767952
rs137901416
rs2293579
rs2538745
rs112394943
rs6669563
rs59409453
rs28502153
rs1957937
rs10773176
rs188918174
rs6881568
rs1985369
rs10840270
rs2967355
rs66731853
rs7164950
rs17677991
rs10845387
rs80324517
rs9419958
rs1611236
rs182059586
rs56061761
rs143190905
rs11699829
rs75664430
rs116863223
rs869785
rs9878436
rs79977579
rs80116508
rs144204502
rs117512405
rs932002
rs450962
rs131797
rs150150565
rs9940099
rs4695407
rs8102497
rs61748181
rs7705526
rs12451892
rs8105767
rs6659669
rs17445108
rs2276182
rs35640778
rs7099229
rs76219171
rs35446936
rs141214782
rs28363070
rs13062095
rs77231040
rs76666449
rs13230646
rs17803849
rs11557154
rs10768683
rs117630647
rs16978028
rs11426156
rs76065543
rs7790856
rs56799554
rs73581419
rs939916
rs3785074
rs11769630
rs6536702
rs6790988
rs3891167
rs11412296
rs142426306
rs2977608
rs77732866
rs11991877
rs4498805
rs78491606
rs9398196
rs6587577
rs202034370
rs11117354
rs611646
rs10112752
rs2555104
rs139669835
rs4731541
rs11212631
rs1332941
rs11584821
rs3093888
rs1291143
rs6776756
rs1003322
rs55747751
rs4724
rs117407747
rs12932179
rs10905255
rs2282764
rs185174247
rs111950327
rs45604339
rs2056726
rs2230590
rs117034449
rs4616688
rs41269079
rs10774624
rs6054257
rs34550383
rs12369950
rs10805346
rs9600019
rs139795227
rs871134
All − MR Egger
All − Inverse variance weighted
−2
−1
0
1
2
MR effect size for
'telomere length || id:ieu−b−4879' on 'IDP_dMRI_ProbtrackX_L1_ptr_r || id:ub
